# Efficacy of artificial femoral head replacement for femoral head avascular necrosis

**DOI:** 10.1097/MD.0000000000015411

**Published:** 2019-04-26

**Authors:** Shou-Feng Wang, Qing-Hui Ji, Xiao-Feng Qiao, Peng Zhao, Yu Xue, Yan-Bao Li

**Affiliations:** a First Ward of Orthopedics Department, First Affiliated Hospital of Jiamusi University; b Department of Orthopedics, Jiamusi Central Hospital; c Department of Surgery, Second Affiliated Hospital of Jiamusi University, Jiamusi; d Department of Orthopedics, Handan Central Hospital, Handan, China.

**Keywords:** artificial femoral head replacement, avascular necrosis, efficacy, femoral head, randomized controlled trial

## Abstract

Background: Femoral head avascular necrosis (FHAN) is one of the most common progressive orthopedic disorders. Previous studies have reported that artificial femoral head replacement (AFHR) can effectively treat patients with FHAN. However, no systematic review has investigated the efficacy of AFHR for FHAN. This study will assess the efficacy of AFHR for patients with FHAN.

Methods: We will search MEDLINE, EMBASE, Web of Science, Cochrane Library, Chinese Biomedical Literature Database, and China National Knowledge Infrastructure up to March 1, 2019 without any restrictions. Any randomized controlled trials for assessing the efficacy of AFHR for patients with FHAN. The methodological quality for each eligible study will be assessed by using Cochrane risk of bias tool. Statistical analysis will be conducted by using RevMan 5.3.

Results: This study will provide current evidence of AFHR for patients with FHAN from several aspects, including pain intensity, function, and limitation of femoral head, health-related quality of life, and safety.

Conclusion: This study will provide latest evidence on assessing the efficacy and safety of AFHR for FHAN.

PROSPERO registration number: PROSPERO CRD42019126249.

## 1 Introduction

Femoral head avascular necrosis (FHAN) is a common disorder of the hip joint.^[1–3]^ If it cannot be treated fairly well, it can frequently cause the damage of femoral head and degradation of hip joints.^[4–5]^ Unfortunately, current treatments still had limited efficacy.^[6–8]^ Furthermore, it can be further aggravated by using steroid, alcoholic intake, sickle cell anemia, metabolic disorders, tumors, or trauma.^[9–10]^ Previous study reported that the incidence of FHAN was about 10,000 to 20,000 new cases in the United States.^[11]^ Impaired blood perfusion and increased intraosseous pressure mainly account for the necrotic process.^[12–13]^ If these conditions cannot be identified at early stages, they may result in osteoarthritis and finally need joint replacement.

Previous clinical trials have reported that joint replacement, especially artificial femoral head replacement (AFHR) can treat this disorder very effectively.^[8,14–26]^ Unfortunately, no study has systematically assessed the efficacy of AFHR for the treatment of FHAN. Therefore, this study will first evaluate the efficacy of AFHR for patients with FHAN.

## 2 Methods

### 2.1 PROSPERO registration

This study has been registered on PROSPERO (CRD42019126249). Its report follows Preferred Reporting Items for Systematic Reviews and Meta-analyses (PRISMA) Protocols statement guidelines.^[27]^

### 2.2 Eligibility criteria for study selection

#### 2.2.1 Types of studies

Randomized controlled trials (RCTs) assessing the efficacy and safety of AFHR for FHAN will be considered for inclusion without any restrictions. Noncontrolled trials, non-RCTs, and quasi-RCTs will be excluded.

#### 2.2.2 Types of participants

All patients who have been diagnosed with FHAN will be included without any limitations of region, race, gender, and so on.

#### 2.2.3 Type of interventions

All patients in the experimental group receive any kinds of AFHR. It will be excluded if the patients receive AFHR with other treatments. All patients in the control group can receive any therapies, but not the AFHR.

#### 2.2.4 Type of outcomes

Primary outcome is pain intensity, as measured by any pain scales, such as visual analog scale. Secondary outcomes include function and limitation of attacked femoral head, as measured by Western Ontario and McMaster Universities Osteoarthritis Index, or any other tools; health-related quality of life, as assessed by 36-Item Short Form Health Survey; and any complications after the surgery.

### 2.3 Search strategy

The following electronic databases of Cochrane Library, MEDLINE, EMBASE, Web of Science, Chinese Biomedical Literature Database, and China National Knowledge Infrastructure will be searched up to March 1, 2019 without any restrictions. In addition, we will also search dissertations, clinical registry, and reference lists of relevant reviews. We will provide search strategy sample for MEDLINE in Table 1. Additionally, identical search strategies will also be built and utilized to other electronic databases.

**Table 1:**
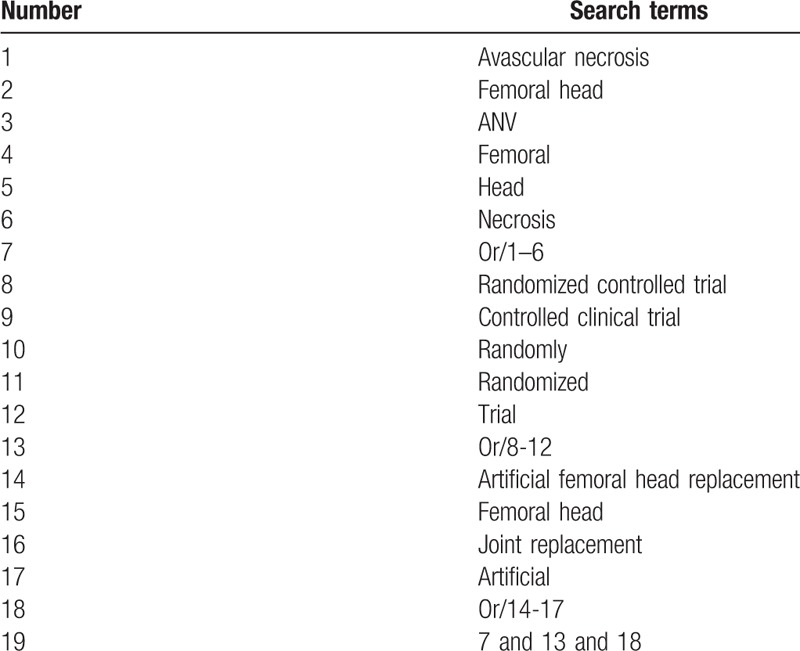
Search strategy applied in MEDLINE database.

### 2.4 Data collection

#### 2.4.1 Study selection

This study selection has 2 phrases. At the first phrase, 2 authors will screen the titles and abstracts of all records independently to exclude studies obviously failing to the eligibility criteria. At second phrase, the full-texts of remaining studies will be read by the same 2 authors to further determine whether they finally meet all eligibility criteria for inclusion. Any disagreements will be solved by a third author through discussion. The results of the study selection process will be presented in the PRISMA flowchart.

#### 2.4.2 Data extraction

Two authors will collect all important information and extract data based on the predesigned data extraction sheet independently. All disagreements will be resolved by judging from a third author through discussion. The extracted information comprises of study details, such as author, country, year of publication, and so on; patient details, such as characteristic data at baseline, diagnostic criteria, and so on; study methods, such as sample size, randomization, blinding, and so on; treatments details in both experimental and control group; and all outcome measurements.

#### 2.4.3 Missing data

If any data are unclear or not reported in the primary study, we will contact the primary authors by email or phone to inquire them if it is possible. Otherwise, we will analyze the available information and will also carry out sensitivity analysis to investigate the potential effect of the missing data.

### 2.5 Methodological quality assessment

Two authors will evaluate the methodological quality for each eligible study according to the standard criteria of Cochrane Collaboration Tool independently. It comprises of 7 aspects, and each aspect will be classified into 3 levels: high, unclear, or low risk of bias. Any disagreements will be solved by a third author through discussion.

### 2.6 Statistical analysis

We will use RevMan 5.3 software to conduct statistical analysis, including data pooled, and meta-analysis performance.

#### 2.6.1 Treatment effect measurement

Continuous data will be recorded as mean difference or standardized mean difference and 95% confidence intervals (CIs). Dichotomous data will be recorded as risk ratio and 95% CIs.

#### 2.6.2 Heterogeneity assessment

Heterogeneity among all eligible studies will be determined by utilizing *I*^2^ test. When *I*^2^ ≤50%, heterogeneity is considered as minor. When *I*^2^ >50%, heterogeneity is considered as significant.

#### 2.6.3 Data synthesis

When heterogeneity is minor, we will use a fixed-effect model to synthesize data and will perform meta-analysis if it is possible. When heterogeneity is significant, we will analyze the causes of the heterogeneity by carrying out subgroup analysis or meta-regression analysis. When heterogeneity is minor after subgroup analysis, we will still pool the data and conduct the meta-analysis. When heterogeneity is still significant, the data will not be pooled, and narrative descriptions will be reported.

#### 2.6.4 Subgroup analysis

When heterogeneity is significant, subgroup analysis will be operated based on the different study characteristics, interventions, and outcomes.

#### 2.6.5 Sensitivity analysis

Sensitivity analysis will be carried out to examine the robustness of data pooled results by taking away low-quality RCTs.

#### 2.6.6 Publication bias

When at least 10 RCTs are included, funnel plot^[28]^ and Egger regression^[29]^ will be used to check if there is publication bias.

## 3 Discussion

FHAN is a very common disorder among elderly population. It greatly affects quality of life for patients with this condition. Previous studies have reported that several managements can help to relieve this disorder. However, the efficacy is not satisfied. Fortunately, joint replacement, especially as for AFHR has been reported to treat this condition fairly well and can significantly improve the quality of life for patients with FHAN. To our best knowledge, this study will firstly and systematically investigate the efficacy of AFHR for patients with FHAN. The results of this study will provide very helpful evidence to determine whether AFHR is an effective management for patients with FHAN. The findings will also summarize helpful evidence for both patients and doctors.

## Author contributions

**Conceptualization:** Shou-Feng Wang, Xiao-Feng Qiao, Peng Zhao, Yu Xue, Yan-Bao Li.

**Data curation:** Shou-Feng Wang, Qing-Hui Ji, Xiao-Feng Qiao, Yan-Bao Li.

**Formal analysis:** Shou-Feng Wang, Qing-Hui Ji.

**Funding acquisition:** Shou-Feng Wang.

**Investigation:** Yan-Bao Li.

**Methodology:** Shou-Feng Wang, Qing-Hui Ji, Xiao-Feng Qiao.

**Project administration:** Yan-Bao Li.

**Resources:** Shou-Feng Wang, Qing-Hui Ji, Xiao-Feng Qiao, Peng Zhao, Yu Xue.

**Software:** Shou-Feng Wang, Qing-Hui Ji, Xiao-Feng Qiao, Peng Zhao, Yu Xue.

**Supervision:** Yan-Bao Li.

**Validation:** Qing-Hui Ji, Yu Xue, Yan-Bao Li.

**Visualization:** Xiao-Feng Qiao, Yan-Bao Li.

**Writing – original draft:** Shou-Feng Wang, Qing-Hui Ji, Xiao-Feng Qiao, Peng Zhao, Yu Xue, Yan-Bao Li.

**Writing – review and editing:** Shou-Feng Wang, Qing-Hui Ji, Peng Zhao, Yu Xue, Yan-Bao Li.
